# Prevalence of Diabetes and Its Relationship With Body Mass Index Among Elderly People in a Rural Area of Northeastern State of India

**DOI:** 10.7759/cureus.12747

**Published:** 2021-01-17

**Authors:** Gajendra K Medhi, Gitashree Dutta, Prasanta Borah, Markordor Lyngdoh, Amitav Sarma

**Affiliations:** 1 Community Medicine, North Eastern Indira Gandhi Regional Institute of Health & Medical Sciences, Shillong, IND; 2 Epidemiology and Nutrition, ICMR-Regional Medical Research Centre, Northeast (NE) Region, Dibrugarh, IND; 3 Anatomy, North Eastern Indira Gandhi Regional Institute of Health & Medical Sciences, Shillong, IND

**Keywords:** elderly, diabetes, bmi, obesity

## Abstract

Background

Diabetes and its complications are a major public health concern in elderly populations. However, there is little population-based data on diabetes and its risk factors among the elderly population living in rural areas of India. The objective of this population-based study was to assess the prevalence of diabetes in the elderly population and its relationship with body mass index (BMI).

Methodology

A population-based, cross-sectional study was conducted among elderly individuals (≥60 years) during the period 2013-2016 in rural areas of Dibrugarh district of Assam. Multi-stage sampling design was adopted to select the study participants. Data on socio-demographic profile and diagnosis/treatment history of diabetes were collected using pre-designed and pre-tested questionnaire. Fasting blood sugar was tested. Weight and height were measured to calculate BMI. Multivariate logistic regression analysis was performed to assess the relationship between diabetes and BMI.

Results

Data were collected from 430 (male: 210, female: 220) individuals. The overall prevalence of diabetes was 7.9% (male: 7.1%, female: 8.6%). Higher level of education was associated with increased prevalence of diabetes. Prevalence of diabetes increased as the BMI of participants increased. Prevalence of diabetes among obese individuals (BMI ≥25 kg/m^2^) was 30.4% compared to only 5% among normal weight individuals. Obesity was associated with eight-fold higher risk of diabetes compared with the individuals of normal weight in multivariate analysis.

Conclusions

The study reveals nearly 8% population-based prevalence of diabetes in rural elderly people in the study district. Our study provides epidemiological evidence that obesity is a major driver of diabetes among rural elderly people.

## Introduction

Diabetes mellitus has steadily evolved as a major public health problem over the past quarter century in India and across the globe [1]. India is contributing a major part of the global diabetes burden, and is considered the diabetic capital of the world [1,2]. India has an estimated 77 million people with diabetes, which is the second highest in the world next to China. Diabetes burden is projected to be 101 million in 2030 and 134 million in 2045 [3]. Population ageing is attributed to be one of the driving forces behind the increase in diabetes prevalence in India [4]. Earlier studies have reported that the prevalence of diabetes mellitus is higher among the elderly compared with middle-aged or young adults [5-7].

Historically a disease of the affluent, recent epidemiological evidence indicates a rising diabetes mellitus incidence and prevalence in urban India’s middle-class and working-class poor sections as well as among the rural populations [6,8]. Studies from different parts of India have provided evidence of the rising prevalence of overweight/obesity in India, and overweight/obesity have been found to be most important contributor of rising prevalence of diabetes in the country [4,6].

Diabetes and its complications may take a major toll on the quality of life of the elderly [9]. Diabetes in the elderly can cause substantial morbidity from macro- and micro-vascular complications, higher mortality, reduced functional status, and increased risk of institutionalization [9-12]. Hence, it is imperative to understand the burden and risk factors of diabetes in the elderly population. However, there is a paucity of population-based data on the prevalence and risk factors of diabetes among the rural elderly population where more than 70% of the population resides, especially in the northeastern parts of India [8]. This study aimed to investigate the prevalence of diabetes among the elderly population in a rural area of a northeastern state of India along with its relationship with body mass index (BMI).

## Materials and methods

A community-based, cross-sectional study was conducted during the period 2013-2016 among elderly individuals aged ≥60 years in rural areas of Dibrugarh district of Assam, India. A multi-stage sampling design was adopted to recruit the participants into the study. In the first stage, two development blocks out of seven were selected randomly from the total list of development blocks in the district. In the last stage, seven villages from each selected blocks were chosen to conduct the study. All community-dwelling individuals 60 years and above were eligible to participate in the study. A total of 430 eligible individuals were recruited in the study. The study protocol was reviewed and approved by the Institutional Ethical Committee of Regional Medical Research Centre (RMRC), Dibrugarh. Informed consent was taken from all the respondents before data collection.

After obtaining informed consent, all the participants were interviewed by trained interviewers using a pre-designed and pre-tested questionnaire through face-to-face interview to collect data on socio-demographic variables and treatment seeking behaviors.

All individuals who were diagnosed as diabetic by a physician, and/or under treatment for diabetes (i.e., insulin and/or oral hypoglycemic agents), and/or individuals who had a fasting blood glucose level of ≥126 mg/dL after an overnight fasting of at least eight hours, and/or two-hour post-glucose value of ≥200 mg/dL were considered to have diabetes [13,14]. Capillary blood glucose (CBG) was measured using a standardized digital glucometer (Accu-Check, Roche diagnostics, Indianapolis, IN, USA) [14]. The blood collection and testing on the spot was carried out as per the guidelines provided in the manual of the company. Blood glucose level in mg/dL was recorded from the monitor.

Measurements of height and weight were done as per the guidelines of the World Health Organization (WHO) [14,15]. The weight was recorded to a minimum of 0.5 kg. Similarly, the height was recorded to a minimum of 0.5 cm.

BMI of participants was calculated using the formula: weight (kg)/height (m^2^). Participants were divided into four groups for both males and females according to the WHO Asia Pacific guidelines: Underweight: BMI <18.5 kg/m^2^; normal weight: BMI 18.5-22.99 kg/m^2^; overweight: BMI 23-24.99 kg/m^2^; and obese: BMI ≥25 kg/m^2^ [16].

Statistical analysis

Data were entered and analyzed using SPSS version 21 (IBM, Armonk, NY, USA). Descriptive statistics such as frequency and mean with standard deviation (SD) were used to present the results. Chi-square test was used for comparison of proportions across groups, and t-test was used to compare continuous variables. Univariate and multivariate binary logistic regression analyses were carried out to produce crude and adjusted odds ratios (ORs) with 95% confidence intervals to examine the relationships between outcome variable (i.e., diabetes) and explanatory variables (i.e., BMI) after adjustment for socio-demographic variables such as age, gender, educational status, and marital status. A p-value of <0.05 was considered statistically significant for all the statistical procedures.

## Results

Table 1 shows the socio-demographic characteristics of the study participants. A total of 430 (male: 210, female: 220) individuals participated in the study. The mean age of the participants was 68.71 (SD: 7.42) years. Most of the participants were in the age group of 60-69 (58.1%) years, 53.4% were currently married (58.6%), and 30.5% had no formal education. Overall, 14.1% of the participants were obese. The overall prevalence of diabetes was 7.9% (n = 34) among the study participants.

**Table 1:** Characteristics of study participants. SD, standard deviation; BMI, body mass index

Variables		n (%)
Age (years)	60-69	250 (58.1)
	70-79	138 (32.1)
	80+	42 (9.8)
	Men age ± SD	68.71 ± 7.42
Gender	Male	210 (48.8)
	Female	220 (51.2)
Educational status	No formal education	131 (30.5)
	Up to high school	224 (52.1)
	Beyond high school	75 (17.4)
Marital status	Married	252 (58.6)
	Widowed	173 (40.2)
	Separated/divorced	2 (0.5)
	Unmarried	3 (0.7)
BMI (kg/m^2^) (N = 399)	Underweight	127 (32)
	Normal weight	161 (40.6)
	Overweight	53 (13.4)
	Obese	56 (14.1)
Diabetes status	Present	34 (7.9)
	Absent	396 (92.1)
Total		430

Among the 34 diabetic participants, 27 were known diabetics and seven were newly diagnosed during the survey.

Table 2 shows the prevalence of diabetes stratified by age, gender, educational, and marital status. No significant gender difference was found in the prevalence. Age trend indicates that diabetes was confined mostly in the age group of 60-69 years, and none of the participants were found to have diabetes in the >80-year age group. The highest educational group (i.e., beyond high school level) had the highest prevalence compared to their less educated counterparts. Married participants had significantly higher prevalence of diabetes than widowed.

**Table 2:** Prevalence of diabetes according to socio-demographic variables.

Variables	Prevalence	P-Value
	n (%)	
Age (years)		0.007
60-69 (n = 250)	25 (10)	
70-79 (n = 138)	9 (6.5)	
80+ (n = 42)	0 (0)	
Gender		0.347
Male (n = 210)	15 (7.1)	
Female (n = 220)	19 (8.6)	
Educational status		0.009
No formal education (n = 131)	8 (4.3)	
Less than high school (n = 224)	3 (4.5)	
Beyond high school (n = 75)	23 (13)	
Marital status		0.05
Married (n = 252)	25 (9.9)	
Widowed (n = 173)	9 (5.2)	

Table 3 presents the mean BMI level according to the diabetes status of the participants. The mean BMI was found to be significantly higher in diabetics than non-diabetics in both males and females.

**Table 3:** Mean BMI (diabetics versus non-diabetics). BMI, body mass index; SD, standard deviation

	Mean BMI ± SD		P-Value
	Diabetics	Non-diabetics	
Male	23.96 ± 4.62	19.97 ± 3.82	0.000
Female	25.35 ± 4.34	20.69 ± 5.1	0.000
Total	24.75 ± 4.45	20.33 ± 4.51	0.000

Table 4 shows that the prevalence of diabetes increased as the BMI level of participants increased. Participants with the lowest BMI (i.e., underweight individuals with BMI of <18.5 kg/m^2^) had the lowest prevalence (2.4%) and those with the highest BMI (i.e., obese individuals with BMI of ≥25 kg/m^2^) had the highest prevalence of diabetes (30.4%).

**Table 4:** Association between diabetes and BMI. BMI, body mass index

BMI categories	Prevalence, n (%)
Underweight	3 (2.4)
Normal weight	8 (5)
Overweight	4 (7.5)
Obese	17 (30.4)
P-value	<0.0001

Table 5 presents the results of univariate and multivariate logistic regression analyses examining the relationship between diabetes and BMI. In the univariate logistic regression analysis, the crude ORs increased with increasing BMI level. A similar pattern was also observed in multiple logistic regression analysis. The adjusted ORs showed that obese individuals (the highest BMI category) had 8.13 times higher risk of having diabetes compared with normal-weight individuals. Although not statistically significant, being overweight was also associated with nearly 1.5-fold higher odds of having diabetes compared with normal-weight individuals.

**Table 5:** Univariate and multivariate logistic regression analyses showing association between diabetes and BMI. BMI, body mass index; COR, crude odds ratio; AOR, adjusted odds ratio **Significant P-value (P < 0.01)

BMI categories	COR (95% CI)	AOR (95% CI)
Underweight	0.463 (0.120-1.78)	0.523 (0.132-2.068)
Normal weight	1 (reference)	1 (reference)
Overweight	1.56 (0.451-5.41)	1.52 (0.425-5.4)
Obese	8.34 (3.35-20.73)**	8.13 (3.19-20.7)**

## Discussion

This population-based, cross-sectional study documents the prevalence of diabetes among the elderly population (>60 years) in a northeastern state of India, and reveals strong epidemiological evidence of association between BMI and diabetes in this population. The overall prevalence of diabetes was 7.9% in this study. There are no population-based data on the prevalence of diabetes among the elderly in Assam for comparison; however, the prevalence documented in this study was higher than the overall prevalence of 4.4% among adult population (>20 years) in Assam found in a recent population-based study [9]. The present prevalence is slightly lower than the national prevalence of about 10% among the rural elderly (>65 years) [9]. However, the prevalence was found to be much lower compared to 23% reported by Medhi et al. among the urban elderly in a recent population-based study conducted in the same district, which reflects existing rural-urban differences in the prevalence of diabetes in India [17]. Our study adds to the limited but growing body of evidence suggesting that diabetes is no longer confined to the urban areas of India but is gradually emerging as a public health concern in rural areas as well [6,8,18]. Considering that over 70% of India’s population resides in rural areas, even a small increase in the prevalence of diabetes in rural areas will imply that a large proportion of individuals will require chronic care as rural populations already suffer with issues such as poor access to health services and poverty [6,8].

Out of 34 total diabetics, 27 were known diabetics and seven (20.5%) were newly diagnosed during the survey, indicating a significant burden of undiagnosed diabetes in the elderly. In an earlier study conducted in Assam, prevalence of undiagnosed diabetes was found to be 50% among adult rural population (>20 years) [6]. Incidental diagnosis of diabetes is generally more common in elderly individuals because of their frequent healthcare contact; hence, prevalence of undiagnosed diabetes is likely to be less in the elderly than younger populations [6,19].

Age-specific prevalence indicates that diabetes was more common among the 60-69-year age group, and none were found to have diabetes in the >80-year age group. Higher prevalence in younger age group probably reflects that diabetes among the elderly in rural population is a relatively recent phenomenon. In this study, no gender difference in the prevalence of diabetes was revealed, which is consistent with findings from other Indian studies [7,8,17,20,21]. However, few studies have documented male preponderance of diabetes [8].

The highest educational group had the highest prevalence of diabetes in this study, which confirms the earlier report from a large multicenter study [5,6]. Such findings suggest that diabetes remains a disease of the more affluent sections of society in the rural areas of India [6]. Higher prevalence in currently married elderly compared with widowed could be due to the younger age of married participants.

It is clear from this study that obesity is an important risk factor of diabetes in this population, which confirms the findings of earlier studies from India [5,6,8,18,20-22]. We found that the prevalence of diabetes increased with increasing BMI level as the prevalence of diabetes was only 2.4% among underweight individuals and 5% among normal-weight individuals which increased to 7.5% among overweight and 30.4% among obese individuals. In multivariate analysis, obesity was associated with more than a 8.3-fold higher risk of diabetes compared with normal-weight individuals. Although not statistically significant, being overweight was also associated with a 1.5-fold higher risk of diabetes compared with normal-weight individuals. Obesity was found to be a strong risk factor in this study, even though we used a lower BMI cut-off value (BMI ≥25 kg/m^2^) to define obesity suggested for Asian populations as opposed to the standard cut-off value (BMI ≥30 kg/m^2^). In general, Indians have a lower BMI than those of Europeans; however, the risk of diabetes increases at very low levels of BMI for Indians [8,16]. As the obesity epidemic is showing an increasing trend in India, there may be further escalation of obesity-related diabetes in this population, warranting greater public efforts to contain the obesity epidemic [23].

Limitations

The study had few limitations. First, as the study is a cross-sectional study, it is difficult to draw causal inferences. Second, various important confounding variables (such as family history of diabetes, dietary, and life-style factors) were not taken into account in the multivariate analysis, which may limit us in elucidating the true relationship between explanatory variables (i.e., BMI) and outcome variables (i.e., diabetes). Third, measurement of CBG was done by a glucometer device instead of venous blood glucose estimation due to logistical issues. Several previous studies have reported that CBG is a feasible alternative for screening in epidemiological studies in which obtaining venous samples might be difficult. Finally, the study was conducted in one district; hence, the findings of the study are not generalizable to the entire region or country.

## Conclusions

The present study provides reliable and recent epidemiological data on the prevalence of diabetes mellitus among the rural elderly population in a representative population in a district of Assam, India. Approximately 8% of the general elderly rural population have diabetes mellitus, indicating that diabetes is emerging as an important public health issue in the rural areas of India, warranting comprehensive public health actions for prevention, screening/diagnosis, and ensuring accessibility to treatment and care to the growing number of diabetics in rural areas. This study also highlights that obesity is a key parameter associated with diabetes mellitus even in the rural populations in India. Given the current increasing trend of obesity in the country, prevalence of obesity-associated diabetes is expected to further increase in the rural population. Weight control programs, particularly among the younger populations, will be cost-effective in addressing the diabetes epidemic. For more effective public health actions, further detailed studies should be conducted to investigate the risk factors of diabetes and obesity among rural populations in India.
